# Effects of Fertilizer Application Intensity on Carbon Accumulation and Greenhouse Gas Emissions in Moso Bamboo Forest–*Polygonatum cyrtonema* Hua Agroforestry Systems

**DOI:** 10.3390/plants13141941

**Published:** 2024-07-15

**Authors:** Huiying Chen, Xuekun Cheng, Xingfa Zhang, Haitao Shi, Jiahua Chen, Ruizhi Xu, Yangen Chen, Jianping Ying, Yixin Wu, Yufeng Zhou, Yongjun Shi

**Affiliations:** 1 State Key Laboratory of Subtropical Silviculture, Zhejiang A&F University, Hangzhou 311300, China; 2 Zhejiang Province Key Think Tank, Institute of Ecological Civilization, Zhejiang A&F University, Hangzhou 311300, China; 3 Key Laboratory of Carbon Cycling in Forest Ecosystems and Carbon Sequestration of Zhejiang Province, Zhejiang A&F University, Hangzhou 311300, China; 4 School of Environmental and Resources Science, Zhejiang A&F University, Hangzhou 311300, China; 5 Forestry Bureau of Qujiang District, Quzhou 324000, China; 6 Agriculture and Rural Bureau of Lin’an District, Hangzhou 311300, China; 7 Forestry and Water Bureau of Longyou County, Quzhou 324000, China

**Keywords:** fertilizer application, agroforestry, Moso bamboo forest, *Polygonatum cyrtonema* Hua, greenhouse gas emissions, carbon accumulation

## Abstract

Agroforestry management has immense potential in enhancing forest carbon sequestration and mitigating climate change. Yet the impact and response mechanism of compound fertilization rates on carbon sinks in agroforestry systems remain ambiguous. This study aims to elucidate the impact of different compound fertilizer rates on soil greenhouse gas (GHG) emissions, vegetation and soil organic carbon (SOC) sinks, and to illustrate the differences in agroforestry systems’ carbon sinks through a one-year positioning test across 12 plots, applying different compound fertilizer application rates (0 (CK), 400 (A1), 800 (A2), and 1600 (A3) kg ha^−1^). The study demonstrated that, after fertilization, the total GHG emissions of A1 decreased by 4.41%, whereas A2 and A3 increased their total GHG emissions by 17.13% and 72.23%, respectively. The vegetation carbon sequestration of A1, A2, and A3 increased by 18.04%, 26.75%, and 28.65%, respectively, and the soil organic carbon sequestration rose by 32.57%, 42.27% and 43.29%, respectively. To sum up, in contrast with CK, the ecosystem carbon sequestration climbed by 54.41%, 51.67%, and 0.90%, respectively. Our study suggests that rational fertilization can improve the carbon sink of the ecosystem and effectively ameliorate climate change.

## 1. Introduction

According to the IPCC Sixth Assessment Report, human-emitted greenhouse gases (GHGs) such as carbon dioxide (CO_2_), methane (CH_4_), and nitrous oxide (N_2_O) have been identified as primary causes of exacerbating global climate warming since the Industrial Revolution [1]. The increasing concentration of these gases in the atmosphere amplifies the greenhouse effect, raising the Earth’s surface temperature. Key sources of GHG emissions include energy production from human activities [2], excessive deforestation, and land use changes [3]. Therefore, effective control of GHG emissions is crucial to combating current global climate change. Forest ecosystems store approximately 45% of terrestrial carbon [4], and are thus vital in the mitigation of global warming [5]. In practical applications, forest management practices such as fertilization, logging, and forest tending can alter soil physico-chemical properties, affect soil GHG emissions, and improve ecosystem carbon sink capacity [6,7].

Currently, over 1500 species of bamboo plants have been documented globally [8,9], primarily found in tropical and subtropical climate zones. China, as the primary origin of bamboo plants, reached 7.527 million ha of bamboo forest area in 2021, with Moso bamboo forests covering 5.277 million ha, constituting roughly 69.78% of the total area [10]. Moso bamboo, one of the most widely distributed bamboo plants with high economic value in southern China [11,12], is an evergreen plant of the gramineae family, which is adaptable to humid environments. It is also a fast-growing bamboo plant [13], allowing it to quickly accumulate a large amount of biomass and carbon storage in a short time. Compared with other slow-growing stands, Moso bamboo is considered to be an ideal indicator for studying the short-term carbon sequestration capacity of ecosystems. As an important arbor species for carbon sequestration, Moso bamboo can absorb a large amount of carbon dioxide during its growth, convert it into organic matter, and store it in the plant, thus helping to alleviate the rise in GHG concentrations in the atmosphere to a certain extent. Moso bamboo is utilized for a multitude of applications, including building materials, furniture manufacturing, pulp production, landscaping, and edible bamboo shoots [14].

Nevertheless, in recent years, the quantity of abandoned bamboo forests has climbed due to the low price of Moso bamboo and the trend of rising labor costs [15]. In light of this situation, we should adopt scientific management measures to exert its ecological benefits. *Polygonatum Cyrtonema* Hua is a medicinal and edible plant from the genus *Polygonatum* in the Liliaceae family. It is commonly grown in acidic soils that are cool and moist [16], which is similar to the growth environment of Moso bamboo. Previous studies have shown that the quality of *Polygonatum* vulgaris tubers can be improved only after they have been grown for more than four years [17]. This means that it is difficult for farmers to realize direct economic benefits from their land in the short term. Apart from its medicinal value, *Polygonatum cyrtonema*’s rhizome system contributes to soil conservation and stability, reducing soil erosion, and thereby maintaining soil biodiversity and ecosystem stability. As the market demand for traditional Chinese medicine supplements increases year by year, the resources of wild *Polygonatum cyrtonema* Hua have been heavily depleted, with its quantity becoming smaller and smaller; therefore, its survival is greatly threatened [18]. In summary, the growth environmental conditions of Moso bamboo and *Polygonatum cyrtonema* Hua are similar. Moso bamboo forest has ample understory space, strong carbon sequestration ability, and good ecological benefits. At the same time, *Polygonatum cyrtonema* Hua has promising economic prospects. Therefore, planting *Polygonatum cyrtonema* Hua under Moso bamboo forest is regarded as a strategy to take into account both ecological and economic benefits.

As a common management measure of the forest ecosystem, the agroforestry management model is considered a sustainable development model [19]. This model provides new ideas for mitigating climate change, improving ecosystem carbon sink capacity, and improving the ecological environment through the rational allocation of agricultural and forestry resources. In addition, the agroforestry management model can also increase soil organic carbon storage and microbial activity, helping to improve forest carbon sequestration capacity [20,21]. Among them, planting *Polygonatum cyrtonema* Hua under Moso bamboo forest is a common agroforestry management model [22]. Previous research has indicated that compound fertilizer, as a commonly used soil amendment, can significantly enhance biomass accumulation, growth, and development of vegetation as well as increase its capacity to sequester carbon when properly applied. Mao et al. [23] observed that applying compound fertilizers can greatly boost the *Pinus massoniana Lamb* seedlings’ biomass accumulation. Applying compound fertilizer when cultivating *Polygonatum cyrtonema* Hua under Moso bamboo forests serves to address its diverse nutrient requirements, foster robust plant growth, and enhance yield and quality, while concurrently sustaining soil fertility and ecological stability [24]. While past studies have mainly concentrated on the influence of fertilization on crop yields and soil carbon storage in agroforestry management systems, the impact on carbon sink function remains an area yet to be thoroughly explored.

We carried out an entire year’s trial in the field in a Moso bamboo forest and *Polygonatum cyrtonema* Hua system in Lin’an, Zhejiang, to better investigate the influence of compound fertilizer intervention intensity on the carbon sink function of agroforestry management systems. This research aims to investigate how varying compound fertilizer application rates make differences in the annual carbon sequestration of soil organic carbon and vegetation, along with the dynamic characteristics of GHG emissions. Our assumptions were as follows: (1) The impact of applying compound fertilizers on soil GHG emissions is not singular, and a certain amount of application can significantly promote soil GHG emissions; (2) the short-term application of compound fertilizers can increase annual vegetation and soil carbon sequestration; and (3) the influence of compound fertilizer on short-term annual carbon sequestration in the ecosystem is contingent upon its application rate. This research may provide a theoretical basis for promoting carbon sequestration in agroforestry management systems.

## 2. Results

### 2.1. Effects of Compound Fertilizer Intervention Intensity on Soil Environmental Factors and Unstable Carbon and Nitrogen Pools

**Figure 1:**
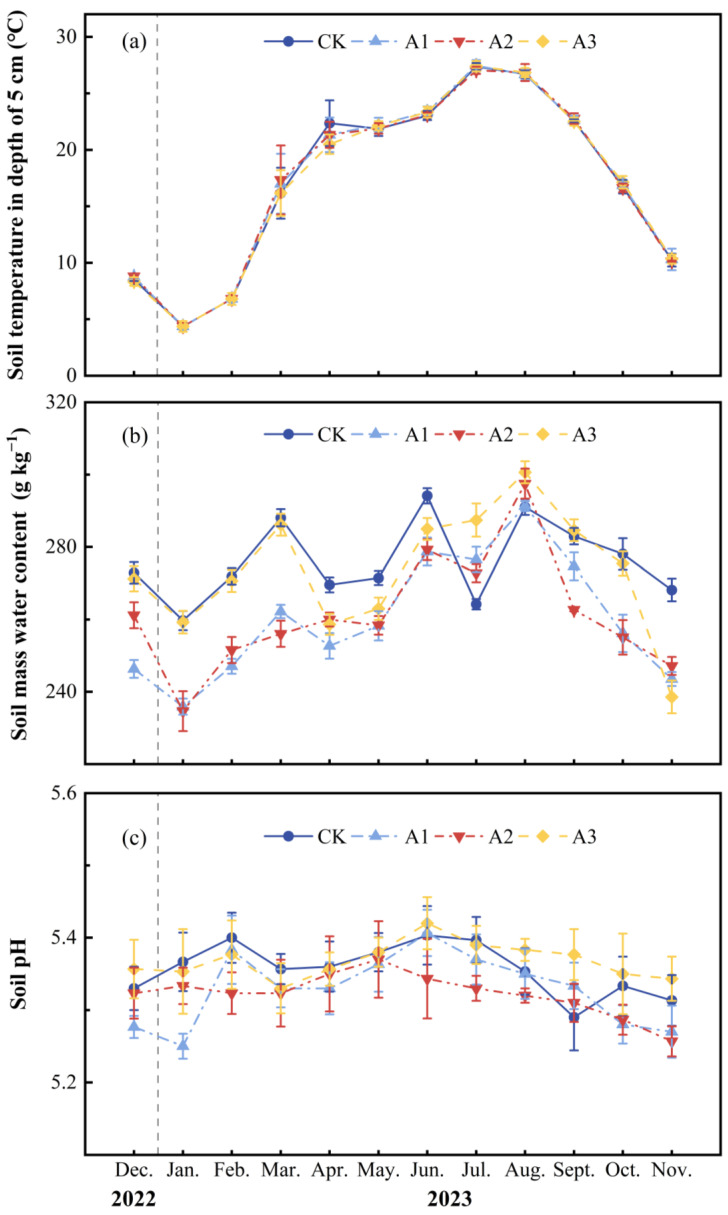
Monthly average ± standard deviation (*n* = 3) at different fertilizer application rates: (**a**) soil temperature, (**b**) soil mass water content, and (**c**) soil pH. The deviations are indicated by error bars.

In a year-long field experiment, the monthly average mass water content was 276.02 ± 2.11, 260.28 ± 0.20, 261.34 ± 1.63, and 273.38 ± 2.01 g kg^−1^ for CK (0 kg ha^−1^), A1 (400 kg ha^−1^), A2 (800 kg ha^−1^), and A3 (1600 kg ha^−1^) (Figure 1b; Table 1), with significant differences between all treatments (*p* < 0.001). Additionally, the fluctuation pattern of 5 cm soil depth temperature in each treatment was highly consistent with the trend of temperature change in the study area (Figure 1a), with the lowest temperature occurring in winter (December to February) and the highest temperature reaching its peak in summer (July to August), without significant differences in soil temperature among treatments (*p* > 0.05). The study results showed that the annual average pH values of CK, A1, A2, and A3 were 5.36 ± 0.03, 5.33 ± 0.02, 5.32 ± 0.03, and 5.37 ± 0.03, respectively (Figure 1c; Table 1), but non-significantly (*p* > 0.05).

**Figure 2:**
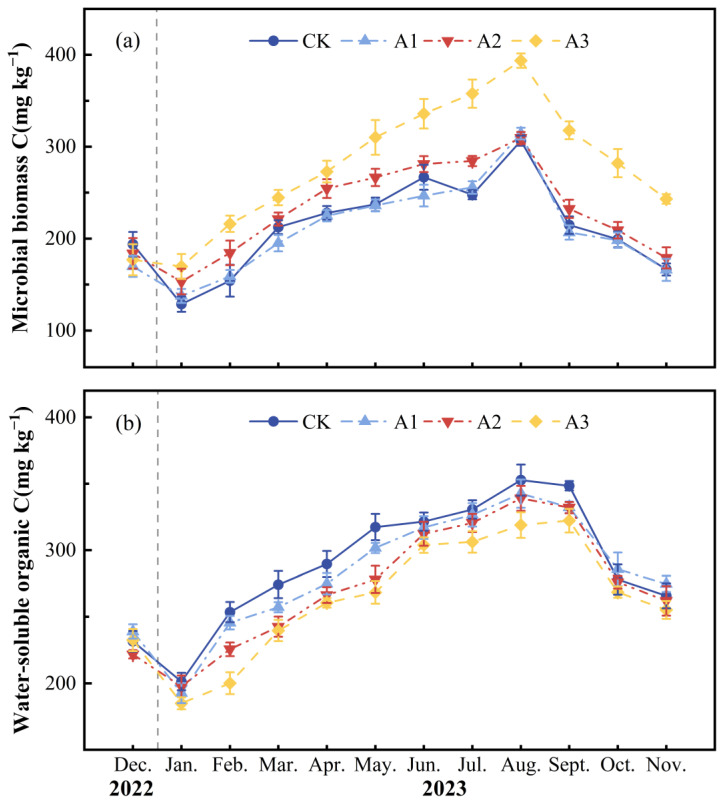
Monthly average ± standard deviation (*n* = 3) at different fertilizer application rates, including (**a**) soil microbial biomass carbon and (**b**) soil water-soluble organic carbon. The deviations are indicated by error bars.

In the soil carbon pool, the monthly mean soil MBC concentration of CK was 213.02 ± 4.75 mg kg^−1^. After applying compound fertilizer, the monthly average MBC contents of A1, A2, and A3 were 209.19 ± 0.95, 229.98 ± 7.73, and 276.76 ± 5.36 mg kg^−1^, respectively (Figure 2a; Table 1), and compared with CK, A1 decreased by 1.80%, while A2 and A3 increased by 7.96% and 29.92%, respectively (*p* < 0.001). The monthly average WSOC concentration of CK was 288.73 ± 4.58 mg kg^−1^. Compared with CK, the monthly average WSOC concentration in A1, A2, and A3 decreased by 2.16%, 5.51%, and 8.76%, respectively, with significant differences (*p* < 0.001) (Figure 2b; Table 1).

**Figure 3:**
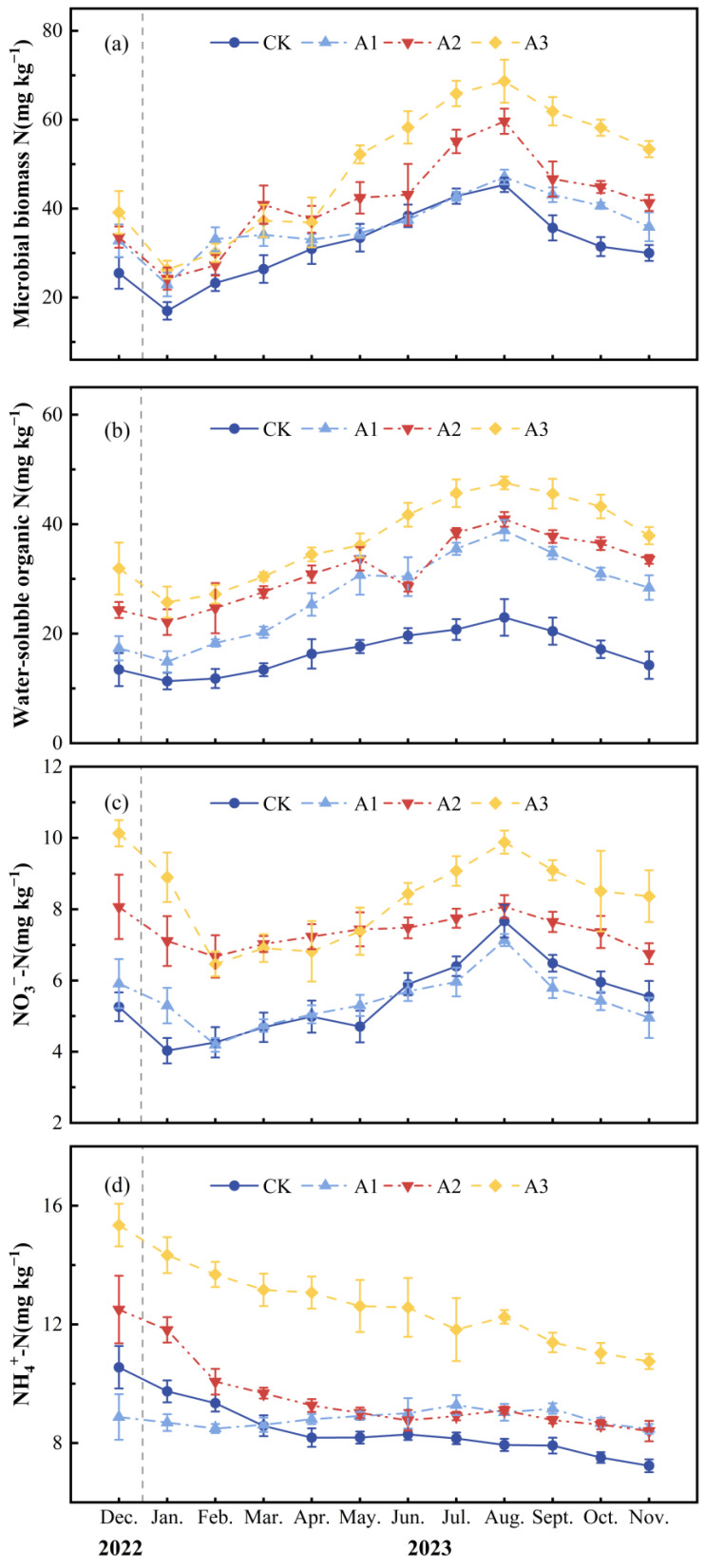
Monthly mean ± standard deviation (*n* = 3) at different fertilizer application rates for (**a**) soil microbial nitrogen, (**b**) soil water-soluble organic nitrogen, (**c**) soil nitrate nitrogen, and (**d**) soil ammonium nitrogen. The deviations are indicated by error bars.

In the soil nitrogen pool, the monthly average soil MBN concentration and monthly average WSON concentration of CK were 31.68 ± 0.29 mg kg^−1^ and 16.59 ± 0.65 mg kg^−1^, respectively. Compared with CK, as the intensity of compound fertilizer intervention increased, the monthly average MBN concentration increased by 15.04%, 30.63%, and 54.69%, respectively (*p* < 0.001) (Figure 3a; Table 1). Additionally, the monthly average WSON concentration also increased by 63.52%, 90.23%, and 124.70%, respectively (*p* < 0.001) (Figure 3b; Table 1). The monthly average NO_3_^−^-N concentration of CK, A1, A2, and A3 were 5.49 ± 0.10, 5.45 ± 0.24, 7.39 ± 0.15, and 8.33 ± 0.19 mg kg^−1^, respectively (*p* < 0.001) (Figure 3c; Table 1). The monthly average NH_4_^+^-N concentration of CK was 8.47 ± 0.12 mg kg^−1^. The monthly average NH_4_^+^-N concentration of A1, A2, and A3 was higher than that of CK by 4.30%, 13.07%, and 49.61%, respectively (*p* < 0.001) (Figure 3d; Table 1).

### 2.2. Effect of Compound Fertilizer Intervention Intensity on Greenhouse Gas Emissions from Agroforestry Management Systems

**Figure 4:**
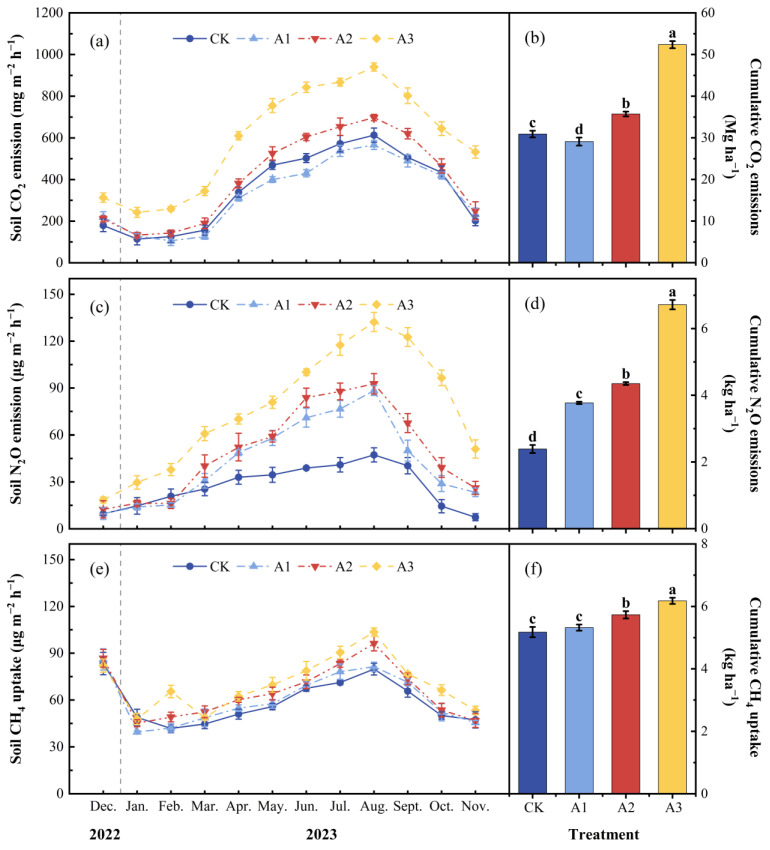
Monthly mean ± standard deviation (*n* = 3) under different fertilization rates: (**a**) soil CO_2_ emission flux, (**c**) soil N_2_O emission flux, (**e**) soil CH_4_ uptake flux, (**b**) soil cumulative CO_2_ emissions, (**d**) soil cumulative N_2_O emissions, and (**f**) soil cumulative CH_4_ uptake. The deviations are represented by error bars, and distinct lowercase letters represent significant variations among different treatments (*p* < 0.001).

The monthly mean soil CO_2_ emission fluxes for CK, A1, A2, and A3 throughout the 12-month field positioning experiment were 351.27 ± 9.47, 330.74 ± 11.17, 406.25 ± 6.64, and 596.18 ± 9.92 mg m^−2^ h^−1^ (Figure 4a; Table 1). Moreover, the monthly average soil CO_2_ emission flux for A1 was significantly 5.84% lower than that of CK (*p* < 0.05), whereas A2 and A3 were significantly greater than CK’s by 15.65% and 69.72%, respectively (*p* < 0.001). CK, A1, A2, and A3 had a total annual flux of soil CO_2_ emissions of 30.90 ± 0.82, 29.10 ± 0.97, 35.72 ± 0.58, and 52.37 ± 0.86 Mg ha^−1^, respectively (Figure 4b).

The monthly average soil N_2_O emission flux and the yearly total soil N_2_O emission flux of CK were 27.28 ± 1.31 µg m^−2^ h^−1^ and 2.39 ± 0.12 kg ha^−1^, respectively. After applying compound fertilizer, the monthly average soil N_2_O emission fluxes of A1, A2, and A3 were 42.90 ± 0.46, 49.47 ± 0.46, and 76.50 ± 1.58 µg m^−2^ h^−1^, respectively (Figure 4c; Table 1). In addition, a notable rise in the yearly total soil N_2_O emission flux was observed in conjunction with an increase in compound fertilizer application (*p* < 0.001). The yearly total soil N_2_O emission flux of A1, A2, and A3 were 3.77 ± 0.04, 4.35 ± 0.04, and 6.72 ± 0.14 kg ha^−1^, respectively. In comparison with CK, the values of A1, A2, and A3 increased significantly by 57.73%, 81.86%, and 180.94%, respectively (*p* < 0.001) (Figure 4d).

According to our experimental results, all four treatments were capable of absorbing CH_4_ from the atmosphere. The monthly average soil CH_4_ absorption flux and annual cumulative CH_4_ absorption flux of CK were 58.94 ± 1.90 µg m^−2^ h^−1^ and 5.18 ± 0.17 kg ha^−1^, respectively. After the application of compound fertilizer, the monthly mean soil CH_4_ absorption flux of A1, A2, and A3 were 60.59 ± 1.10, 65.26 ± 1.37, and 70.46 ± 1.11 µg m^−2^ h^−1^, respectively (Figure 4e; Table 1). Compared with CK, the annual cumulative CH_4_ absorption flux of A1, A2, and A3 significantly increased by 2.79%, 10.70%, and 19.34%, respectively (*p* < 0.001) (Figure 4f).

In summary, our research indicated that compared with the control group (CK), the total soil GHG flux significantly increased when the application rate of compound fertilizer was 800(A2) and 1600(A3) kg ha^−1^ (*p* < 0.001); however, when the intervention strength of compound fertilizer was 400 (A1) kg ha^−1^, the total soil GHG emission flux decreased, but not significantly (*p* > 0.05).

### 2.3. Environmental Soil Factors’ Impact on Soil Greenhouse Gas Emissions

The outcomes of stepwise regression analysis indicated a significant association between soil GHG emissions and soil environmental variables. Specifically, soil temperature, soil WSON, soil WSOC, and soil MBC concentrations indicated a notable positive association with the soil CO_2_ emission flux (Table 2, Table 3 and Table 4). Regardless of the treatment with any application rate, there was no discernible link between soil MBN content and soil CO_2_ emission flux (Table 2). In all treatments, soil temperature, soil NH_4_^+^-N, soil MBC, and WSON concentrations showed a significant positive association with soil N_2_O emission flux. A significant correlation was not identified between soil NO_3_^−^-N concentration, soil pH and mass water content, and soil N_2_O emission flux (Table 3). In all treatments, mass water content, soil NH_4_^+^-N, and NO_3_^−^-N concentration had a remarkable positive correlation with CH_4_ absorption flux, and CH_4_ absorption flux was not significantly impacted by soil pH (Table 4).

In the CK treatment, soil temperature was identified as the positive driver for both soil CO_2_ and N_2_O emission fluxes (Table 2 and Table 3). Under the A1 and A2 treatments, soil NO_3_^−^-N concentration positively influenced both soil CO_2_ emission flux and CH_4_ absorption flux (Table 2 and Table 4). In the A3 treatment, mass water content negatively correlated with soil CO_2_ emission flux (Table 2), while soil WSOC positively affected both soil CO_2_ and N_2_O emission fluxes (Table 2 and Table 3). Additionally, mass water content was positively correlated with CH_4_ absorption flux (Table 4).

**Figure 5:**
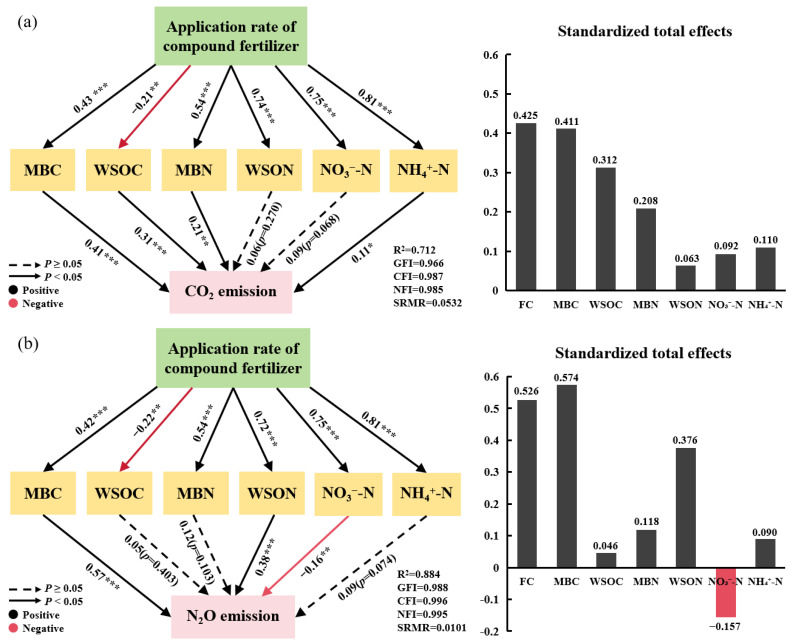

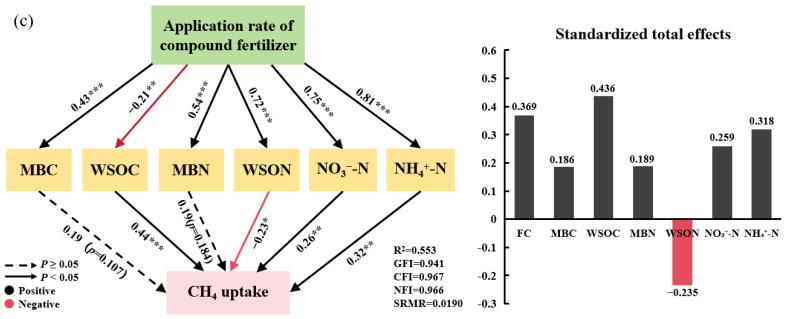
The impacts of compound fertilizer application, soil MBC, WSOC, MBN, WSON, NO_3_^−^-N, and NH_4_^+^-N on the soil: (**a**) CO_2_, (**b**) N_2_O emission flux, and (**c**) CH_4_ absorption flux are illustrated by the structural equation model (SEM), either directly or indirectly. CK, A1, A2, and A3 signify compound fertilizer application rates of 0, 400, 800, and 1600 kg ha^−1^, respectively. The picture on the right presents the standardized total effect diagram corresponding to greenhouse gases, describing the overall impact of different factors on greenhouse gas emissions, where FC denotes compound fertilizer application. The numbers next to the arrows in the structural equation model represent the standardized path coefficients and significance levels. The symbols *, **, and *** signify *p*-values of <0.05, <0.01, and <0.001, respectively. Black and red arrows represent positive as well as negative correlations, whereas solid and dashed arrows indicate significant and non-significant relationships. R^2^ represents the model interpretation rate. The goodness-of-fit index is signified by GFI, the comparative fit index by CFI, the normative fit index by NFI, and the standardized root mean square residual by SRMR.

The structural equation model (SEM) can explain 71.2% and 88.4% of the variation in soil CO_2_ and N_2_O emission fluxes, respectively, as well as 55.3% of the variation in CH_4_ absorption flux (Figure 5a–c). The standardized total effects of the SEM signified that the primary contributors of soil CO_2_ emission were soil MBC concentration (0.411) and WSOC concentration (0.312) (Figure 5a), the major contributors of soil N_2_O emission flux were soil MBC concentration (0.574) and soil WSON concentration (0.376) (Figure 5b), and soil WSOC concentration (0.436) and soil ammonium nitrogen (NH_4_^+^-N) concentration (0.318) were the primary contributors of soil CH_4_ absorption flux (Figure 5c).

### 2.4. Effects of the Intensity of Compound Fertilizer Intervention on the Carbon Concentrations of Polygonatum Cyrtonema Hua

**Figure 6:**
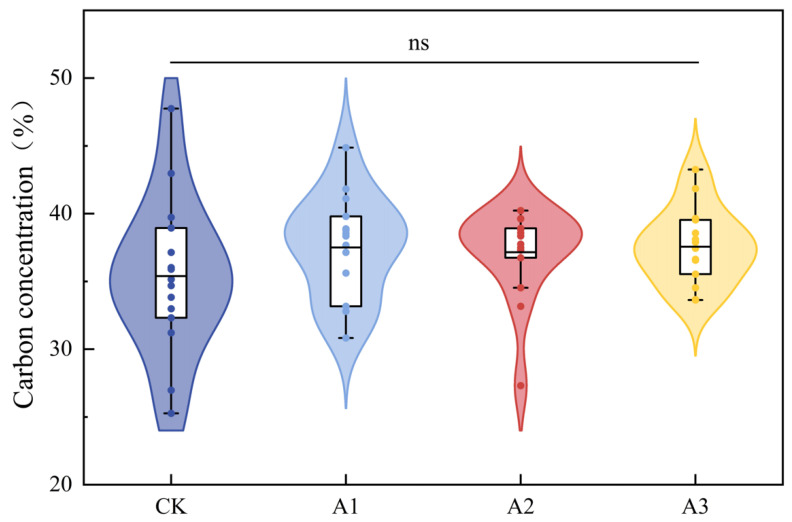
The violin box plot describes the carbon content distribution of 15 *Polygonatum cyrtonema* Hua samples in each treatment in the form of a curve. The horizontal line inside the box denotes the mean value, the upper and lower lines of the box plot indicate the data’s maximum and minimum values, and the upper and lower bounds of the box signify the upper and lower quartiles of data. The acronym “ns” means there are no appreciable differences among the four treatments (*p* > 0.05).

In our study, the intensity of compound fertilizer intervention did not significantly influence the carbon content of *Polygonatum cyrtonema* Hua (*p* > 0.05). The average *Polygonatum cyrtonema* Hua carbon concentrations of CK, A1, A2, and A3 were 35.39 ± 5.70, 37.50 ± 3.82, 37.16 ± 3.11, and 37.56 ± 2.77%, respectively. Figure 6 signified that the carbon concentration of *Polygonatum cyrtonema* Hua was mostly distributed between 33% and 40%. Furthermore, in our study, *Polygonatum cyrtonema* Hua has not yet reached the harvesting stage, so this indicator was not included in the calculation of ecosystem carbon sequestration.

### 2.5. Effects of Compound Fertilizer Intervention Intensity on Vegetation and Soil Carbon Sequestration

We observed no significant difference in the annual carbon sequestration of herbs and shrubs under different treatments (*p* > 0.05) (Table 5). Nevertheless, a notable discrepancy was found in the annual carbon sequestration of the above-ground Moso bamboo (*p* < 0.001) (Table 5). The annual values of carbon sequestered by vegetation were 31.48 ± 1.60, 37.16 ± 1.34, 39.90 ± 0.74, and 40.50 ± 0.95 Mg CO_2_-eq ha^−1^, respectively, when compound fertilizer (CK) was not used, and 400 kg ha^−1^ (A1), 800 kg ha^−1^ (A2), and 1600 kg ha^−1^ (A3) when compound fertilizer was applied. The yearly carbon sequestration amount of vegetation increased significantly with the intensity of compound fertilizer intervention (*p* < 0.001) (Table 5). Compared with not applying compound fertilizer (CK), the annual soil carbon sequestration of A1, A2, and A3 also significantly increased by 32.57%, 42.30%, and 43.30%, respectively (*p* < 0.05) (Table 5), indicating a positive effect of compound fertilizer application on soil carbon sequestration.

The total soil GHG values of CK, A1, A2, and A3 were 31.48 ± 0.81, 30.09 ± 0.96, 36.87 ± 0.58, and 54.22 ± 0.89 Mg CO_2_-eq ha^−1^, respectively. Significant statistical differences were found among the four treatments (*p* < 0.001) (Table 5). In our research, the greater the intervention strength of compound fertilizer, the greater the vegetation and soil carbon sequestration. It is worth noting, however, that it is not the case that the greater the amount of fertilizer used, the greater the yearly carbon sequestration in the ecosystem. Compared with CK, the A1 and A2 ecosystems’ annual carbon sequestration increased significantly by 54.42% and 51.64%, respectively (*p* < 0.01), while the A3 ecosystem’s annual carbon sequestration increased by 0.90%, but not significantly (*p* > 0.05) (Table 5).

## 3. Discussion

### 3.1. Soil GHG Emissions Respond to the Intensity of Compound Fertilizer Intervention

In terrestrial ecosystems, the main process through which soil releases carbon into the atmosphere is through soil CO_2_ emissions [25]. This study showed that the introduction of compound fertilizer in the Moso bamboo forest–*Polygonatum cyrtonema* Hua agroforestry management model may not necessarily increase the soil CO_2_ emission flux. Particularly, the utilization of 400 kg ha^−1^ of compound fertilizer resulted in a 5.82% decrease in yearly cumulative soil CO_2_ emissions (*p* < 0.05) (Figure 4b). This phenomenon may be attributed to the application of lower amounts of compound fertilizers. In this case, microorganisms in the soil may preferentially decompose exogenous organic matter, thereby inhibiting the mineralization process of the original organic matter in the soil, leading to the reduction in soil CO_2_ emissions. This is in alignment with the outcomes observed by Yu et al. [26] in cornfield trials. During the experiment, adding 800 and 1600 kg ha^−1^ of compound fertilizers significantly increased soil CO_2_ emissions, which could be explained as follows: soil CO_2_ emission flux was closely related to soil temperature and soil MBC concentration, which is identical to previous research by scholars [27] (Figure 5a). Compound fertilizer application may improve the soil microorganisms’ living environment by raising the soil surface’ nutrient content, stimulating the reproduction of microorganisms in the soil and the increase in microbial biomass. This further increases the activity of soil microorganisms, which causes them to secrete more soil enzymes, and soil enzyme activity is significantly positively correlated with soil MBC concentration [28] (Figure 2a), indirectly affecting soil CO_2_ emissions. Higher microbial activity accelerates the mineralization of soil organic matter [29], thereby increasing soil CO_2_ emissions. Another reason may be that during the growth process, plants secrete phytohormones to promote root development, and the roots of Moso bamboo and *Polygonatum cyrtonema* Hua may release organic matter into the soil. These secretions provide additional carbon sources and nutrients for soil microorganisms [29], which may stimulate plant root respiration and soil heterotrophic respiration [30]. Finally, the total soil respiration rate was enhanced and soil CO_2_ emissions were promoted.

N_2_O exhibits a global warming potential 300 times that of CO_2_ [31]. In this study, compound fertilizer intervention significantly increased soil N_2_O emission flux throughout the entire experimental period, which is in conformity with previous research findings [32]. Structural equation modeling and stepwise regression analysis showed that changes in soil labile carbon, nitrogen pools, and environmental factors directly or indirectly affect soil N_2_O emissions (Table 3, Figure 5b). Soil N_2_O emissions are primarily affected by nitrification (aerobic process) and denitrification (anaerobic process) [33,34]. Hydrothermal conditions and soil physical and chemical properties have an important impact on the production of N_2_O by microorganisms [35]. Specifically, compound fertilizers are high in nitrogen. Therefore, adding more compound fertilizers to the soil may increase the amount of nitrogen present, resulting in stimulating the metabolic activity and reproduction of microorganisms, which may enhance the rates of soil nitrification and denitrification, thus promoting soil N_2_O emissions. Another reason may be changes in labile nitrogen pools in the soil. After utilizing compound fertilizer, the concentrations of NO_3_^−^-N and NH_4_^+^-N in the soil significantly rise, which acts as a substrate source for microbial nitrification and denitrification [36], thus promoting nitrification and denitrification of the soil and causing soil’s emissions of N_2_O to rise (Figure 3c,d and Figure 4c,d). Additionally, the rise in MBC and MBN concentrations in the soil promotes the increase in the number of microorganisms, thereby promoting N_2_O emissions from the soil (Figure 2a, Figure 3a and Figure 4c,d).

This study demonstrated that the intervention of compound fertilizer can significantly reduce soil CH_4_ emissions. Methanotrophic bacteria are important CH_4_ consumers in the soil, and soil CH_4_ flux is influenced by the activity of these bacteria and soil physicochemical properties. Previous research has indicated that compared to monoculture, agroforestry systems typically improve soil structure, which is conducive to gas diffusion and soil drainage [36], potentially enhancing the proliferation and activity of methane-oxidizing bacteria within the soil. This process results in the oxidation of CH_4_ to CO_2_ and H_2_O, thereby reducing soil CH_4_ emissions. Another reason may be that compound fertilizer intervention increases soil organic carbon storage. Existing studies have shown a beneficial correlation between soil CH_4_ uptake rate and soil organic matter content [37], indicating that soil rich in organic matter is more conducive to CH_4_ uptake. It can be inferred that compound fertilizer intervention can directly increase soil WSOC and NH_4_^+^-N concentrations, thereby indirectly promoting CH_4_ uptake based on the results of structural equation modeling and stepwise regression linear models (Table 4, Figure 5c). However, some studies have shown that fertilization can increase soil CH_4_ emissions. He et al. [38] found, for instance, that using organic fertilizer instead of chemical fertilizer significantly reduced soil CO_2_ emissions but increased CH_4_ and N_2_O emissions. In a paddy rotation experiment, Lou et al. [39] found that adding green manure raised soil CO_2_ and CH_4_ emissions. These differences may be attributed to variations in research subjects, types and amounts of fertilizers used, and experimental designs.

It is essential to note that the observation period of this study was only one year, which may not be sufficient to fully capture the long-term effects of compound fertilizer intervention on soil GHGs. Previous studies have demonstrated that adding nitrogen fertilizers over an extended period did not increase soil GHGs [40]. In contrast, Zhang et al. [41] found in field experiments that long-term fertilization increased GHG emissions. Therefore, in future studies, it would be beneficial to extend the experimental period to further investigate the effects of fertilizer intervention on greenhouse gas emissions.

### 3.2. The Annual Carbon Sequestration of Vegetation, Soil, and Ecosystem Carbon Accumulation Respond to the Intensity of Compound Fertilizer Intervention

Vegetation carbon pools play an essential role in the agroforestry ecosystem. The three nutrients—nitrogen (N), phosphorus (P), and potassium (K)—necessary for the growth of Moso bamboo were provided by applying compound fertilizer in this study. Field positioning test findings demonstrated that there was a notable positive correlation between the annual carbon sequestration amount of vegetation and the amount of compound fertilizer applied (*p* < 0.001). This phenomenon may be attributed to fertilization promoting the growth and photosynthesis of Moso bamboo. As the intensity of compound fertilizer intervention increases, the number of Moso bamboo plants also increases correspondingly, resulting in an increase in Moso bamboo biomass. However, it is worth noting that when the intervention of compound fertilizer exceeds 400 kg ha^−1^, the growth rate of vegetation carbon sequestration gradually slows down, which may imply a certain saturation effect of the intensity of compound fertilizer intervention on vegetation carbon sinks. In addition, the underground rhizomes of *Polygonatum cyrtonema* Hua contain a large amount of carbon, but this study found that there is no significant relationship between carbon content and the intensity of compound fertilizer intervention (*p* > 0.05) (Figure 6). Therefore, the carbon sequestration by *Polygonatum cyrtonema* Hua underground rhizomes was not included in the calculation of annual carbon sequestration by vegetation.

Soil organic carbon storage is a significant function that influences ecosystem carbon sinks. Compound fertilizer intervention can significantly increase soil organic carbon storage (*p* < 0.001) (Table 5), which is in line with the research of Chen et al. [42]. The main reason for this increase in carbon storage may be that the intervention of compound fertilizers can significantly increase soil organic matter content. Meanwhile, Moso bamboo and *Polygonatum cyrtonema* Hua gradually shed their aging leaves during their growth, which may cause litter to accumulate and rapidly decompose. The decomposition of litter temporarily stores organic carbon [43], thereby increasing soil organic carbon storage. After the intervention of compound fertilizer, microbial activity in the soil increased, promoting the fixation of more carbon in the atmosphere. And the stability of soil aggregates was significantly enhanced, which promoted plant growth and root development, thus enhancing soil carbon’s fixations [44,45]. In addition, in the absence of fertilization treatment (CK), the possible reason for the large changes in soil organic carbon storage within a year was the spatial heterogeneity of soil [46]. In December 2022, we randomly selected three points near each static box to conduct soil sampling with a manual shovel. After sampling, we buried the sampling points to preserve the original state of the sample sites and reduce soil disturbance. When the soil was sampled for the second time in November 2023, the sampling point may have changed because one year had passed. Although the basic conditions of the sample plots are basically the same, changes in sampling points would lead to changes in soil nutrients. Therefore, soil organic carbon changes greatly at different times and locations. Although this is a short-term experiment, it provides an important foundation and inspiration for subsequent research. Therefore, in subsequent research, when soil sampling is conducted, the sampling points should be marked so that the sampling points before and after are consistent to eliminate the impact of soil heterogeneity. At the same time, the frequency of soil sampling should be increased, and the sampling interval should be monthly or quarterly. Moreover, measurement and analysis should be conducted to eliminate errors and identify objective patterns. Furthermore, our research site is located in the southeast coastal area of China, which may also lead to certain uncertainties and regional limitations in the conclusions drawn from our research.

## 4. Materials and Methods

### 4.1. Experimental Region

**Figure 7:**
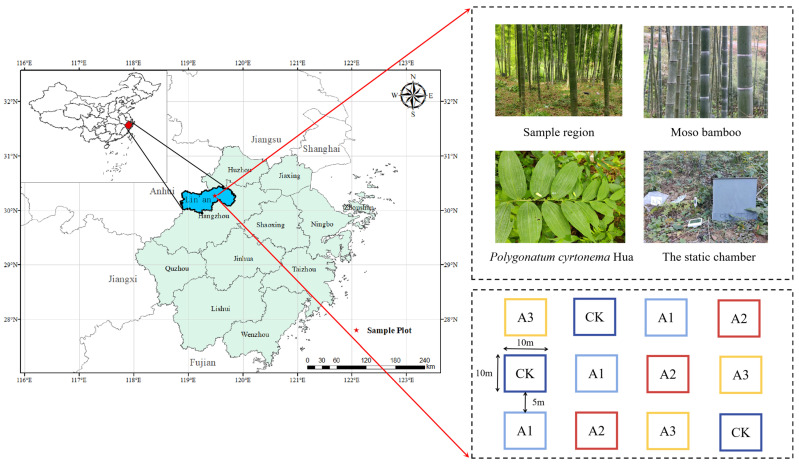
Location and design of experiments (CK, A1, A2, and A3 were applied with 0, 400, 800, and 1600 kg ha^−1^ of compound fertilizer, respectively).

**Figure 8:**
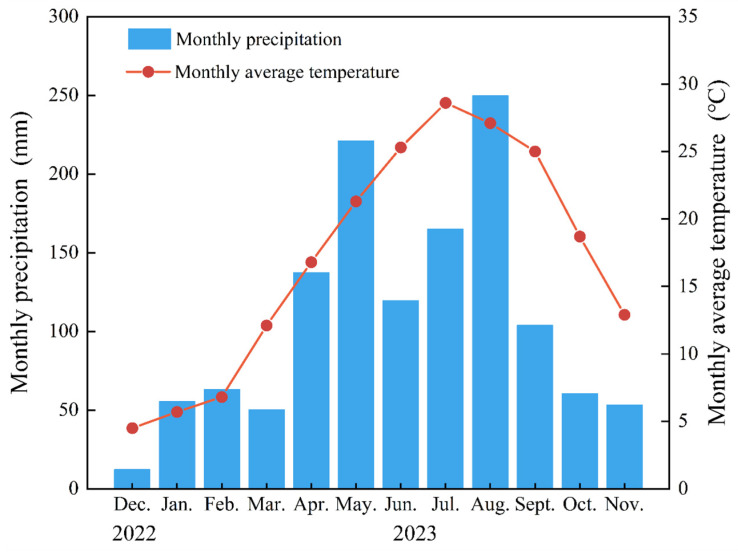
Mean monthly temperature and mean monthly precipitation throughout the experiment period.

Sui Mei Forestry Field, Lin’an District, Zhejiang Province, China is the location of the survey region (119°84′ E, 30°23′ N) (Figure 7). It features a terrain characterized by low mountains and hills, with an altitude of approximately 85 m and a slope of approximately 25°. This area enjoys the typical warm, humid subtropical monsoon climate, with plenty of sunshine and precipitation. Annual average precipitation ranges between 1250 and 1500 mm, with a yearly mean temperature of 17.1 °C, 158 precipitation days, and a yearly average frost-free period of 237 days. The soil in the survey region is classified as Ferralsols according to the 2014 FAO soil classification system [47]. The average monthly temperature and precipitation during the experimental period are displayed in Figure 8.

In November 2022, before the start of fertilization treatment, 0–20 cm of surface soil was collected and litter was removed. The soil samples were transported to the laboratory for basic determination of physical and chemical properties. The basic soil properties were as follows: soil bulk density 0.91 g cm^−3^, pH 5.32, soil organic carbon (SOC) 28.32 g kg^−1^, K available 61.3 mg kg^−1^, P available 6.8 mg kg^−1^, and alkali-hydrolyzable nitrogen 121.78 mg kg^−1^.

### 4.2. Experimental Design

In November 2020, pure Moso bamboo forest sample plots with nearly identical growth and site conditions were selected. We planted *Polygonatum cyrtonema* Hua seedlings in these sampling plots to transform the pure Moso bamboo forest into an agroforestry management model. The plant spacing of the *Polygonatum cyrtonema* seedlings was 20 cm × 20 cm. A random block design was adopted, with 4 treatments and 3 repetitions per treatment, and a total of 12 test plots were planted, each with an area of 10 m × 10 m. To prevent interference from Moso bamboo underground roots on adjacent plots, a 5 m isolation zone was established between each trial plot. Winter is an important season for the root growth of *Polygonatum cyrtonema* Hua; therefore, in the third year of the agroforestry management model (November 2022), we applied compound fertilizer (mainly composed of N, P, and K compounds: N, 15%; P_2_O_5_, 15%; and K_2_O, 15%) and spread it evenly on the sample plot. The treatments were as follows: (1) control group, no compound fertilizer (CK) was applied; (2) application of 400 kg ha^−1^ of compound fertilizer (A1); (3) application of 800 kg ha^−1^ of compound fertilizer (A2); and (4) application of 1600 kg ha^−1^ of compound fertilizer (A3). In November 2022, the sample plots were weeded. Following that, a stationary box was positioned at the diagonal junction of every trial area for GHG collection for an extended period of time. Sampling and analysis began one month after fertilization and the frequency of GHG collection and soil sample collection was once a month. The trial lasted for 12 consecutive months (December 2022 to November 2023).

### 4.3. Measurements of Soil GHG Emissions

GHG samples from experimental plots were gathered monthly and subjected to static chamber gas chromatography analysis between December 2022 and November 2023. The static box was constructed from polyvinyl chloride (PVC) panels and had three parts: a base that measured 30 cm long, 30 cm wide, and 10 cm high; a top section that featured a U-shaped channel that measured 5 cm wide and 5 cm high; and a removable cover that was 30 cm long, 30 cm wide, and 10 cm high. The base was embedded 10 cm deep into the soil for stability. The sample collection of each static box was carried out between 9:00 and 11:00 on a sunny morning at the beginning of every month. Before gathering gas samples, we first filled the U-shaped groove at the top of the base of the static box with water, and then filled the static box with water. All the vegetation was cut off at the root with scissors, a fan was placed in the middle of the base to facilitate the mixing of air within the box, and finally, a lid with a rubber stopper on the top was put on to form a sealed space in the entire box. At 0, 10, 20, and 30 min after sealing the static box, we used a 100 mL syringe to insert a rubber stopper with the removable cap to collect samples four times. Simultaneously, a thermometer with a button (iButton DS1925-F5, Wdsen Electronic Technology Corporation, LTD, Shanghai, China) was embedded 5 cm deep close to the static box to obtain the soil temperature. Ultimately, the collected gas samples were inserted into 100 mL vacuum bags made of aluminum foil (Dalian Bright Chemical Design Institute, Dalian, China) and stored before being returned to the laboratory to be tested for GHG using gas chromatography (GC-2014, Shimadzu Corporation, Tokyo, Japan). After collecting the gas, we removed the lid of the static box and placed it near the base.

The flux of each GHG was computed using Equation (1) [21]:

(1) $$F_{x}=\rho \times \frac{V}{A}\times \frac{273.15}{T}\times {\frac{P}{P}}_{0}\times \frac{{dC}_{t}}{d_{t}}$$

where $F_{x}$ depicts the emission flux or uptake flux of the soil GHGs (N_2_O: µg m^−2^ h^−1^, CH_4_: µg m^−2^ h^−1^, CO_2_: mg m^−2^ h^−1^); ρ depicts the density of the GHG under standard conditions (N_2_O, CH_4_, and CO_2_ are 1.964 × 10^3^, 7.163 × 10^2^, and 1.98 × 10^3^ g m^−3^, respectively); *V* and *A* depict the volume of the static box (m^3^) and the bottom area of the static box (m^2^), respectively; *T* depicts the static box’s temperature within the sampling period; *P* and $P_{0}$ depict the absolute air pressure under standard conditions and the pressure in the static box during the sampling period, respectively; and $\frac{{dC}_{t}}{d_{t}}$ depicts the slope of the change in gas concentration over time per unit time (ppm h^−1^).

The annual cumulative soil GHG emission flux was calculated using Equation (2) [48]:

(2) $$E_{x}=\left(t_{m+1}-t_{m}\right)\times \frac{\sum \left(F_{m+1}+F_{m}\right)}{2}\times 24\times 10^{-5}$$

where $E_{x}$ depicts the yearly cumulative emission flux or uptake flux of the soil GHGs (N_2_O: kg ha^−1^ year^−1^, CO_2_: Mg ha^−1^ year^−1^, and CH_4_: kg ha^−1^ year^−1^); *t* depicts the sampling times; *m* depicts the sampling number; and *F* depicts the monthly soil GHG emission or uptake flux (mg m^−2^ h^−1^).

To better assess the contribution of various GHGs to climate warming, we uniformly converted the emission fluxes of CO_2_, N_2_O, and CH_4_ to CO_2_ equivalents and used Equation (3) to calculate the total GHG emission flux [27]:

(3) $${GHG}_{T}=298E_{N_{2}O}+E_{CO_{2}}-25E_{CH_{4}}$$

where ${GHG}_{T}$ depicts the total soil GHG emission flux (Mg CO_2_-eq ha^−1^); $E_{N_{2}O}$, $E_{{CO}_{2}}$, and $E_{{CH}_{4}}$ depict the yearly cumulative emission fluxes of N_2_O and CO_2_ and the yearly cumulative uptake flux of CH_4_, respectively. On a one-century scale, the warming potential coefficients of N_2_O and CH_4_ converted into CO_2_ equivalents are 298 and 25, respectively [49].

### 4.4. Soil Sampling Collection and Physico-Chemical Property Analyses

During the period from December 2022 to November 2023, while collecting GHG samples every month, three sampling points were randomly selected around each static box, and a manual spade was used to dig into the surface soil profile (0–20 cm), and the soil sample was mixed evenly before collection. Litter on the soil surface was removed before collection to ensure sample purity. When taken back to the laboratory, we crushed larger particles in the soil (but not gravel) with a grinding rod, and picked out gravel larger than 2 mm; next, the soil samples were subjected to a sieving process through a sieve with a hole diameter of 2 mm to obtain the active fraction. Then, we divided the sieved soil sample into two parts: the first part was stored in a 4 °C refrigerator, and soil mass water content (M), soil nitrate nitrogen (NO_3_^−^-N), soil microbial biomass carbon (MBC), soil water-soluble organic carbon (WSOC), soil microbial biomass nitrogen (MBN), soil water-soluble organic nitrogen (WSON), and soil ammonium nitrogen (NH_4_^+^-N) were determined within four days; the second part was naturally air-dried indoors for a month before being analyzed for soil pH and further screened through a 0.15 mm sieve for the analysis of SOC concentrations. The purpose of using two sieves (first through a 2 mm sieve, and then through a 0.15 mm sieve) to measure the soil SOC concentration was to better remove gravel and roots in the soil sample. We compared and analyzed the experimental results of the one-time sieving method and the two-time sieving method; the difference in SOC results between the two sieving methods was not significant, R^2^ is 0.9762 (Figure S1; Table S1).

The mass water content was measured by placing collected soil samples in a natural-draft drier at 105 °C for a full day, and then conducting calculations according to quality changes [50]. The soil pH was estimated using the glass electrode method [51]. The ring knife method was utilized to determine the bulk density of the soil. The concentrations of the available potassium (AK), available phosphorus (AP), and alkali-hydrolyzable nitrogen (AN) were determined by using the NH_4_OAc extraction-flame photometric method, NH_4_F-HCl extraction-colorimetric method, and alkaline hydrolytic diffusion method, respectively [50]. The amounts of soil WSOC and soil WSON were determined using the Total Organic Carbon Analyzer (TOC-V_CPH_, Shimadzu Corporation, Tokyo, Japan) in accordance with Wu et al.’s method [52]. The MBC and MBN concentrations were obtained using the chloroform fumigation method noted by Vance et al. [53]. The indophenol blue colorimetric approach and the ultraviolet spectrophotometric approach were used, respectively, to estimate the concentrations of NH_4_^+^-N and NO_3_^−^-N [54].

In December 2022 and November 2023, air-dried surface (0–20 cm) soil samples were passed through a sieve with a hole diameter of 0.15 mm and analyzed for SOC. The high-temperature external heating potassium dichromate oxidation volumetric method was utilized to determine the amount of SOC present in the sieved soil samples [29], and Equation (4) was to calculate the SOC stocks [55]:

(4) $$C_{SOC}=\sum_{i}C\times D_{i}\times B_{i}\times (1-\omega )\times 100^{-1}$$

where $C_{SOC}$ depicts 0–20 cm soil organic carbon storage (Mg C ha^−1^), i depicts the layer of soil, C depicts concentrations of soil organic carbon (g kg^−1^), $D_{i}$ depicts the soil thickness of the i-th layer (cm), $B_{i}$ depicts the soil bulk density (g cm^−3^), and ω depicts the mass fraction (%) of stone oak, roots, and other organisms with a diameter greater than 2 mm.

The annual carbon sequestration of soil organic carbon was calculated using Equation (5) [27]:

(5) $$\Delta_{SOC}=\frac{44}{12}\times \left(C_{SOC,2023}-C_{SOC,2022}\right)$$

where $\Delta_{SOC}$ depicts the annual soil carbon sequestration (CO_2_-eq Mg ha^−1^ year^−1^); $C_{SOC,2023}$ and $C_{SOC,2022}$ depict soil organic carbon storage in 2023 and 2022, respectively.

### 4.5. Determinations of Vegetation Carbon Storage

In this study, vegetation carbon storage comprises Moso bamboo, shrub, and herbaceous carbon storage, excluding litter carbon stocks. In December 2022 and November 2023, we determined the diameter at breast height (DBH) of each Moso bamboo on each of the twelve plots using a DBH ruler and calculated their biomass based on the single plant biomass calculation model [56]. At the same time, ground shrubs and herbs were collected using the harvesting approach. The specific method was to set up two shrub quadrats of 2 m × 2 m and two herb quadrats of 1 m × 1 m in each plot and collect all shrubs and herbs in the quadrat. Subsequently, the harvested shrubs and herbs were weighed and taken to the laboratory to dry at 105 °C until their weight remained constant. Ultimately, the biomass of shrubs and herbs was calculated using their respective root-to-shoot ratios and then multiplied by the average carbon content of the shrubs and herbs to determine their carbon reserves.

Equations (6) and (7) were employed to estimate the above-ground biomass of an individual Moso bamboo plant as well as its biomass carbon storage per unit area [56]:

(6) $$B_{m}=747.787\times {D_{B}}^{2.771}\times {\left(\frac{0.148A}{0.028+A}\right)}^{5.555}+3.772$$

(7) $$C_{B}=10\times \sum B_{m}\times \left(1+R_{M}\right)\times \frac{C_{F}}{A_{P}}$$

where $B_{m}$ depicts the biomass of a single Moso bamboo plant (kg), $D_{B}$ depicts the DBH of Moso bamboo (cm), A depicts the age class of Moso bamboo, and m depicts the quantity of Moso bamboo plants. $C_{B}$ depicts the biomass carbon storage per unit area of Moso bamboo (Mg C ha^−1^), $R_{M}$ depicts the biomass root-to-stem ratio of Moso bamboo (0.47), $C_{F}$ depicts the average carbon content rate of Moso bamboo (0.5042) [56], and $A_{P}$ depicts the sample plot area (here is 100 m^2^).

Equations (8)–(10) were used to calculate the carbon storage of shrub and herbaceous biomass [57]:

(8) $$B_{SH}=\frac{1}{2}\times \sum_{m=1}^{2}W_{Fm}\times \left(1-\frac{W_{Fm}-W_{Dm}}{W_{Fm}}\right)$$

(9) $$C_{S}=B_{SH}\times \left(1+R_{S}\right)\times C_{SH}\times 20^{-2}$$

(10) $$C_{H}=B_{SH}\times \left(1+R_{H}\right)\times C_{SH}\times 10^{-2}\;$$

where $B_{SH}$ depicts the mean biomass of shrubs or herbs (g), $W_{Fm}$ and $W_{Dm}$ depict the fresh and dry weight (g) of the sample, respectively; the biocarbon stocks of shrubs and herbs (Mg C ha^−1^) are signified by $C_{S}$ and $C_{H}$; $R_{S}$(0.5732) and $R_{H}$(1.58) depict the root-to-shoot ratio of shrubs and herbs, respectively [57,58]; and $C_{SH}$(0.5) depicts the average carbon content of shrubs or herbs.

Vegetation carbon storage and vegetation annual carbon sequestration were calculated using Equations (11) and (12) [58]:

(11) $$C_{V}=C_{B}+C_{S}+C_{H}$$

(12) $$\Delta_{V}=\frac{44}{12}\times \left(C_{V,2023}-C_{V,2022}\right)$$

where $C_{V}$ depicts vegetation carbon storage (Mg C ha^−1^), which is the total carbon storage of Moso bamboo, shrubs, and herbaceous vegetation in the sample plot; $\Delta_{V}$ depicts the annual carbon sequestration of vegetation (CO_2_-eq Mg ha^−1^ year^−1^); and $C_{V,year}$ depicts the vegetation carbon storage (Mg C ha^−1^) in the respective year.

### 4.6. Determination of Carbon Concentrations of Polygonatum Cyrtonema Hua

Rhizome samples of *Polygonatum cyrtonema* Hua were gathered in November 2023; 15 samples were obtained from every treatment, for an altogether total of 60 samples. After collection, the sample’s fresh weight was weighed promptly, and the samples were subsequently sent to the laboratory to be dried at 85 °C in a ventilated drying oven until the weight remained constant; then, the dry weight was determined. To ensure its uniformity and stability, the dried rhizome was divided for crushing processing. Subsequently, the sample was passed through a sieve with a pore size of 0.15 mm and mixed evenly. Ultimately, approximately 3 mg of the sample was weighed and the organic carbon content was measured using an elemental analyzer.

### 4.7. Determination of the Ecosystem Carbon Sequestration

The annual carbon sink of the ecosystem in this study was calculated using Equation (13) [58]:

(13) $$E_{total}=\Delta_{SOC}+\Delta_{V}-{GHG}_{T}$$

where $E_{total}$ depicts the ecosystem’s yearly sequestration of carbon under agroforestry management systems (CO_2_-eq Mg ha^−1^ year^−1^).

### 4.8. Statistics and Analyses

In this study, data collected underwent statistical analysis using SPSS 27.0, Microsoft Excel 2016, and SPSS Amos 26.0, among other software, while Origin 2024 was employed for chart creation. One-way analysis of variance (ANOVA) and least significant difference analysis (LSD) were employed to compare the effects of compound fertilizer intervention intensity on the yearly cumulative soil GHG emission flux, soil carbon sink, and vegetation carbon sink of the agroforestry management system, with a significance level of 0.05. Before data processing, all treatments underwent variance consistency and normality testing using the Kolmogorov-Smirnov test in SPSS 27.0. At the same time, Microsoft Excel 2016 was used for dimensionless processing of all data to eliminate the comparison bias caused by different dimensions and to more accurately compare the differences between different treatments. The stepwise regression method in SPSS 27.0 was employed to analyze the correlation between the environmental factors, the soil’s physical and chemical characteristics, and the soil GHG emission flux. SPSS Amos 26.0 was used to perform a structural equation model (SEM) to analyze the direct as well as indirect effects of fertilizer application on GHG emissions. The following metrics were adopted to evaluate the model fitness: standardized root mean square residual (SRMR < 0.08), comparative fit index (CFI > 0.9), goodness of fit index (GFI > 0.9), and normed fit index (NFI > 0.9).

## 5. Conclusions

Our conclusions are as follows: Regarding soil greenhouse gas emissions, compound fertilizer intervention made a positive difference in soil N_2_O emissions and CH_4_ absorption. At application rates of 800 and 1600 kg ha^−1^, compound fertilizer had a positive effect on soil CO_2_ emissions. It is noteworthy that at an application rate of 400 kg ha^−1^, there was little impact on soil CO_2_ emissions, and no significant difference was found. This was consistent with our hypothesis (1). There was a remarkable positive correlation between the intervention strength of compound fertilizer and soil organic carbon storage as well as vegetation carbon sequestration, which validates our hypothesis (2). Overall, compound fertilizer intervention increased the carbon sink of the ecosystem of A1, A2, and A3 by 54.41%, 51.67%, and 0.90%, respectively, which confirms hypothesis (3). Therefore, in agroforestry management, it is advisable to use compound fertilizer reasonably to minimize soil GHG emissions while enhancing capacity for vegetation carbon sequestration and soil carbon sink, thus achieving sustainable development of agroforestry management. In future research, further investigation can be conducted on the types and amounts of fertilizers, alterations in soil microbial community structure and function after fertilization, and the impact of litter on the ecosystem in order to comprehensively explore the underlying mechanisms of ecosystem carbon sink function.

## Figures and Tables

**Table 1:** The monthly average values of soil temperature, mass water content, pH, labile soil carbon, and nitrogen pools under various treatment conditions; the standard deviation value of annual average greenhouse gas emission flux under different treatment conditions, and among different treatments; and ANOVA results and significant differences between treatments.

Treatment	T (°C)	M (g kg^−1^)	pH	CO_2_ Emission (mg m^−2^ h^−1^)	N_2_O Emission (μg m^−2^ h^−1^)	CH_4_ Uptake (μg m^−2^ h^−1^)	MBC (mg kg^−1^)	WSOC (mg kg^−1^)	MBN (mg kg^−1^)	WSON (mg kg^−1^)	NO_3_^−^-N (mg kg^−1^)	NH_4_^+^-N (mg kg^−1^)
CK	17.23 ± 0.25 a	276.02 ± 2.11 a	5.36 ± 0.03 a	351.27 ± 9.47 d	27.28 ± 1.31 d	58.94 ± 1.90 c	213.02 ± 4.75 c	288.73 ± 4.58 a	31.68 ± 0.29 c	16.59 ± 0.65 d	5.49 ± 0.10 c	8.47 ± 0.12 c
A1	17.31 ± 0.12 a	260.28 ± 0.20 b	5.33 ± 0.02 a	330.74 ± 11.17 c	42.90 ± 0.46 c	60.59 ± 1.10 c	209.19 ± 0.95 c	282.48 ± 1.85 a	36.44 ± 1.06 c	27.14 ± 0.21 c	5.45 ± 0.24 c	8.83 ± 0.18 c
A2	17.25 ± 0.27 a	261.34 ± 1.63 b	5.32 ± 0.03 a	406.25 ± 6.64 b	49.47 ± 0.46 b	65.26 ± 1.37 b	229.98 ± 7.73 b	272.83 ± 2.51 b	41.38 ± 1.91 b	31.57 ± 0.18 b	7.39 ± 0.15 b	9.58 ± 0.12 b
A3	17.15 ± 0.21 a	273.38 ± 2.01 a	5.37 ± 0.03 a	596.18 ± 9.92 a	76.50 ± 1.58 a	70.46 ± 1.11 a	276.76 ± 5.36 a	263.42 ± 4.28 c	48.38 ± 1.32 a	37.29 ± 0.29 a	8.33 ± 0.19 a	12.67 ± 0.30 a
Analysis of variance between treatments	ns	***	ns	***	***	***	***	***	***	***	***	***

Note: CK, A1, A2, and A3 signify compound fertilizer application rates of 0, 400, 800, and 1600 kg ha^−1^, respectively. T signifies soil temperature at a depth of 5 cm; M signifies mass water content; MBC and MBN, respectively, denote soil microbial biomass carbon and soil microbial biomass nitrogen; WSOC and WSON signify soil water-soluble organic carbon and soil water-soluble organic nitrogen; and NO_3_^−^-N and NH_4_^+^-N, respectively, indicate soil nitrate nitrogen and soil ammonium nitrogen. The lowercase letters contained in the standard values ± standard deviation signify significant differences between various treatments as determined via the least significant difference (LSD) test, where *p* = 0.05. ns, and *** signify *p* > 0.05, and *p* < 0.001.

**Table 2:** Under the CK, A1, A2, and A3 treatments, which correspond to compound fertilizer application rates of 0, 400, 800, and 1600 kg ha^−1^, respectively, a stepwise regression analysis model was conducted to explore a link between soil CO_2_ emission flux and soil variables including soil temperature (T, °C), pH, mass water content (M, g kg^−1^), soil water-soluble organic carbon and nitrogen (WSOC, WSON, mg kg^−1^), soil microbial biomass carbon (MBC, mg kg^−1^), as well as soil nitrate nitrogen and ammonium nitrogen (NO_3_^−^-N, NH_4_^+^-N, mg kg^−1^). Standardized coefficients were used as the model’s coefficients. The degree of freedom was 36; *** indicates *p* < 0.001.

GHG	Treatment	Model	df	R^2^	*p*
CO_2_	CK	Y = 0.904T	36	0.812	***
		Y = 0.546T + 0.429WSON	36	0.866	***
	A1	Y = 0.912WSON	36	0.826	***
		Y = 0.812WSON + 0.283NH_4_^+^-N	36	0.895	***
		Y = 0.740WSON + 0.193NH_4_^+^-N + 0.192NO_3_^−^-N	36	0.913	***
		Y = 0.589WSON + 0.141NH_4_^+^-N + 0.204NO_3_^−^-N + 0.195T	36	0.921	***
	A2	Y = 0.945WSOC	36	0.890	***
		Y = 0.888WSOC + 0.154NO_3_^−^-N	36	0.909	***
		Y = 0.892WSOC + 0.142NO_3_^−^-N + 0.120pH	36	0.921	***
	A3	Y = 0.940MBC	36	0.880	***
		Y = 0.507MBC + 0.486WSOC	36	0.928	***
		Y = 0.602MBC + 0.486WSOC−0.158M	36	0.944	***

**Table 3:** Under the CK, A1, A2, and A3 treatments, which correspond to compound fertilizer application rates of 0, 400, 800, and 1600 kg ha^−1^, respectively, a stepwise regression analysis model was conducted to examine a link between soil N_2_O emission flux and soil variables including soil temperature (T, °C), soil water-soluble organic carbon and nitrogen (WSOC, WSON, mg kg^−1^), soil microbial biomass carbon (MBC, mg kg^−1^), as well as soil nitrate nitrogen and ammonium nitrogen (NO_3_^−^-N, NH_4_^+^-N, mg kg^−1^). Standardized coefficients were used as the model’s coefficients. The degree of freedom was 36; *** indicates *p* < 0.001.

GHG	Treatment	Model	df	R^2^	*p*
N_2_O	CK	Y = 0.854T	36	0.721	***
		Y = 0.985T + 0.232NH_4_^+^-N	36	0.752	***
		Y = 0.495T + 0.315NH_4_^+^-N + 0.585WSOC	36	0.797	***
	A1	Y = 0.945MBC	36	0.890	***
		Y = 0.627MBC + 0.353T	36	0.911	***
	A2	Y = 0.940T	36	0.880	***
		Y = 0.639T + 0.339WSON	36	0.902	***
		Y = 0.319T + 0.330WSON + 0.353MBC	36	0.918	***
	A3	Y = 0.937MBC	36	0.873	***
		Y = 0.617MBC + 0.358WSOC	36	0.898	***
		Y = 0.384MBC + 0.314WSOC + 0.212NO_3_^−^-N	36	0.912	***

**Table 4:** Under the CK, A1, A2, and A3 treatments, which correspond to compound fertilizer application rates of 0, 400, 800, and 1600 kg ha^−1^, respectively, a stepwise regression analysis model was conducted to investigate a link between soil CH_4_ absorption flux and mass water content (M, g kg^−1^), soil water-soluble organic carbon and nitrogen (WSOC, WSON, mg kg^−1^), soil microbial biomass carbon (MBC, mg kg^−1^), as well as soil nitrate nitrogen and ammonium nitrogen (NO_3_^−^-N, NH_4_^+^-N, mg kg^−1^). Standardized coefficients were used as the model’s coefficients. The degree of freedom was 36; *** indicates *p* < 0.001.

GHG	Treatment	Model	df	R^2^	*p*
CH_4_	CK	Y = 0.616NO_3_^−^-N	36	0.361	***
		Y = 0.884NO_3_^−^-N + 0.599NH_4_^+^-N	36	0.646	***
		Y = 0.619NO_3_^−^-N + 0.633NH_4_^+^-N + 0.406MBC	36	0.728	***
	A1	Y = 0.712NO_3_^−^-N	36	0.493	***
		Y = 0.508NO_3_^−^-N + 0.323T	36	0.544	***
	A2	Y = 0.830M	36	0.679	***
		Y = 0.654M + 0.357NO_3_^−^-N	36	0.772	***
		Y = 0.768M + 0.283NO_3_^−^-N + 0.244NH_4_^+^-N	36	0.819	***
		Y = 0.518M + 0.211NO_3_^−^-N + 0.501NH_4_^+^-N + 0.478WSOC	36	0.855	***
	A3	Y = 0.683WSON	36	0.451	***
		Y = 0.485WSON + 0.410M	36	0.571	***
		Y = 0.383WSON + 0.381M + 0.272NO_3_^−^-N	36	0.623	***

**Table 5:** The carbon sequestration of different carbon pools (Mg CO_2_-eq ha^−1^) and ecosystem carbon sequestration (Mg CO_2_-eq ha^−1^) under CK (0 kg ha^−1^), A1 (400 kg ha^−1^), A2 (800 kg ha^−1^), and A3 (1600 kg ha^−1^) treatments.

	CK	A1	A2	A3
Herb carbon sequestration	0.91 ± 0.07 a	0.70 ± 0.22 a	0.80 ± 0.21 a	0.96 ± 0.49 a
Shrub carbon sequestration	0.58 ± 0.07 a	0.52 ± 0.04 a	0.64 ± 0.18 a	0.59 ± 0.24 a
Moso bamboo carbon sequestration	29.99 ± 1.52 c	35.93 ± 1.31 b	38.47 ± 1.01 a	38.95 ± 1.41 a
Vegetation carbon sequestration	31.48 ± 1.60 c	37.16 ± 1.34 b	39.90 ± 0.74 a	40.50 ± 0.95 a
Cumulative soil CO_2_ emission	30.90 ± 0.82 c	29.10 ± 0.97 d	35.72 ± 0.58 b	52.37 ± 0.86 a
Cumulative soil N_2_O emission	0.71 ± 0.04 d	1.12 ± 0.01 c	1.30 ± 0.01 b	2.00 ± 0.04 a
Cumulative soil CH_4_ uptake	0.13 ± 0.01 c	0.13 ± 0.01 c	0.14 ± 0.01 b	0.15 ± 0.01 a
Total GHG emissions	31.48 ± 0.81 c	30.09 ± 0.96 c	36.87 ± 0.58 b	54.22 ± 0.89 a
$\Delta_{SOC}$	32.36 ± 2.31 b	42.90 ± 5.36 a	46.04 ± 8.61 a	46.37 ± 3.68 a
Ecosystem carbon sequestration	32.35 ± 3.07 b	49.96 ± 4.94 a	49.07 ± 7.51 a	32.65 ± 3.15 b

Note: Lowercase letters in the same horizontal line indicate a significant difference test (LSD), with statistical significance (*p* < 0.05, *n* = 3).

## Data Availability

The raw/processed data required to reproduce these findings cannot be shared at this time as the data also form part of an ongoing study.

## Supplementary Materials

The following supporting information can be downloaded at https://www.mdpi.com/article/10.3390/plants13141941/s1, Figure S1. The ordinate indicates the SOC concentration measured after the soil sample passes directly through a 0.15 mm sieve. The abscissa represents the SOC concentration measured after the soil sample first passed through a 2 mm sieve and then through a 0.15 mm sieve; Table S1. Results of SOC determination by two methods.
