# Supplementary material for: Effects of Fertilizer Application Intensity on Carbon Accumulation and Greenhouse Gas Emissions in Moso Bamboo Forest–Polygonatum cyrtonema Hua Agroforestry Systems
Source: Plants (Basel). 2024 Jul 15;13(14):1941. doi: 10.3390/plants13141941 (PMC11280953; doi:10.3390/plants13141941)
Supplement: Supplementary file 1 [file plants-13-01941-s001.zip › plants-3004624-supplementary.pdf]

*Comparative analysis of the impact of two soil sample screening methods on SOC results*

In order to verify the impact of soil samples sieved once and sieved twice on the SOC results and eliminate experimental bias, we collected soil in the experimental plot in May 2024, and used the two sieving methods to conduct SOC determination and comparative analysis on 15 soil samples. As can be seen from Figure A1 and Table A1, there is no significant difference on results between the two methods, the $R^2$ of the linear fitting curve of the two sets of data was 0.9762, So when measuring SOC, both screening methods are feasible.

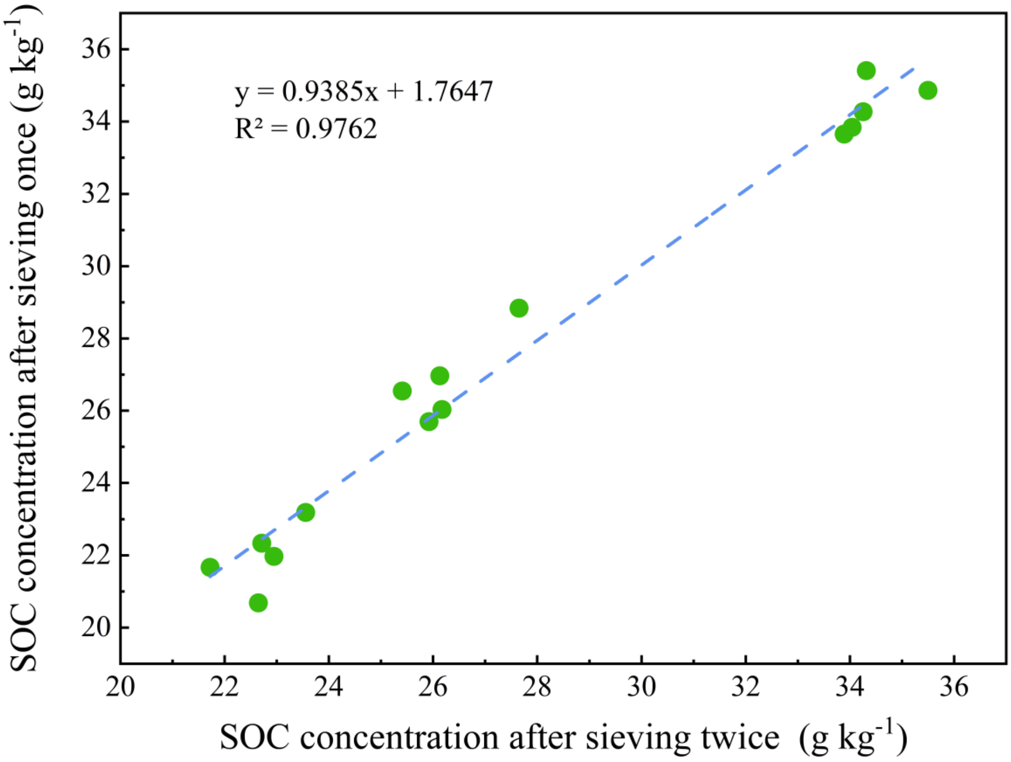


**Figure S1.** The ordinate indicates the SOC concentration measured after the soil sample passes directly through a 0.15 mm sieve. The abscissa represents the SOC concentration measured after the soil sample first passed through a 2 mm sieve and then through a 0.15 mm sieve.

**Table S1.** Results of SOC determination by two methods

| Treatment | Number | Weight of air dried soil (g) | $FeSO_4$ Consumption (ml) | SOC (g $kg^{-1}$) | Treatment | Number | Weight of air dried soil (g) | $FeSO_4$ Consumption (ml) | SOC(g $kg^{-1}$) |
|---|---|---|---|---|---|---|---|---|---|
| blank test | | 0.1220 | 20.1 | | | | | | |
| After sieving once | 1 | 0.1288 | 13.5 | 33.66 | After sieving twice | 1 | 0.1434 | 12.7 | 33.89 |
| | 2 | 0.1303 | 13.3 | 34.27 | | 2 | 0.0997 | 14.9 | 34.25 |
| | 3 | 0.1262 | 13.4 | 34.87 | | 3 | 0.1147 | 13.9 | 35.50 |
| | 4 | 0.1020 | 14.6 | 35.41 | | 4 | 0.1110 | 14.3 | 34.31 |
| | 5 | 0.1242 | 13.7 | 33.84 | | 5 | 0.1196 | 13.9 | 34.04 |
| | 6 | 0.1388 | 15.2 | 23.18 | | 6 | 0.1171 | 15.9 | 23.55 |
| | 7 | 0.1235 | 15.9 | 22.33 | | 7 | 0.1330 | 15.5 | 22.71 |
| | 8 | 0.1182 | 16.2 | 21.67 | | 8 | 0.1270 | 15.9 | 21.72 |
| | 9 | 0.1106 | 16.4 | 21.97 | | 9 | 0.1202 | 15.9 | 22.95 |
| | 10 | 0.1270 | 16.1 | 20.68 | | 10 | 0.1160 | 16.1 | 22.65 |
| | 11 | 0.1380 | 14.7 | 25.70 | | 11 | 0.1140 | 15.6 | 25.92 |
| | 12 | 0.1207 | 14.8 | 28.84 | | 12 | 0.1140 | 15.3 | 27.65 |
| | 13 | 0.1311 | 14.8 | 26.55 | | 13 | 0.1189 | 15.5 | 25.41 |
| | 14 | 0.1242 | 15.0 | 26.97 | | 14 | 0.1131 | 15.6 | 26.13 |
| | 15 | 0.1236 | 15.2 | 26.03 | | 15 | 0.1330 | 14.8 | 26.17 |

Notes: The same horizontal line represents a group of processes.
